# CryoEM reveals BIN1 (isoform 8) does not bind to single actin filaments *in vitro*

**DOI:** 10.17912/micropub.biology.000404

**Published:** 2021-06-04

**Authors:** Zuoneng Wang, Carsten Mim

**Affiliations:** 1 Royal Technical Institute (KTH), Dept. for Biomedical Engineering and Health Systems, Stockholm, Sweden

## Abstract

Cells change their appearance by a concerted action of the cytoskeleton and the plasma membrane. The machinery that bends the membrane includes Bin/Amphiphysin/Rvs (BAR) domain proteins. Recently BAR domain proteins garnered attention as actin regulators, either by recruiting actin regulating proteins or through binding to actin directly. BIN1 (an important protein in Alzheimer’s Disease, heart disease and cancer) is one of the few BAR proteins that bind to actin directly. Here, we imaged a complex of BIN1 and actin with cryoEM. Our results reveal that BIN1 cannot be found on single actin filaments.

**Figure 1. BIN1 interaction with actin filaments:**
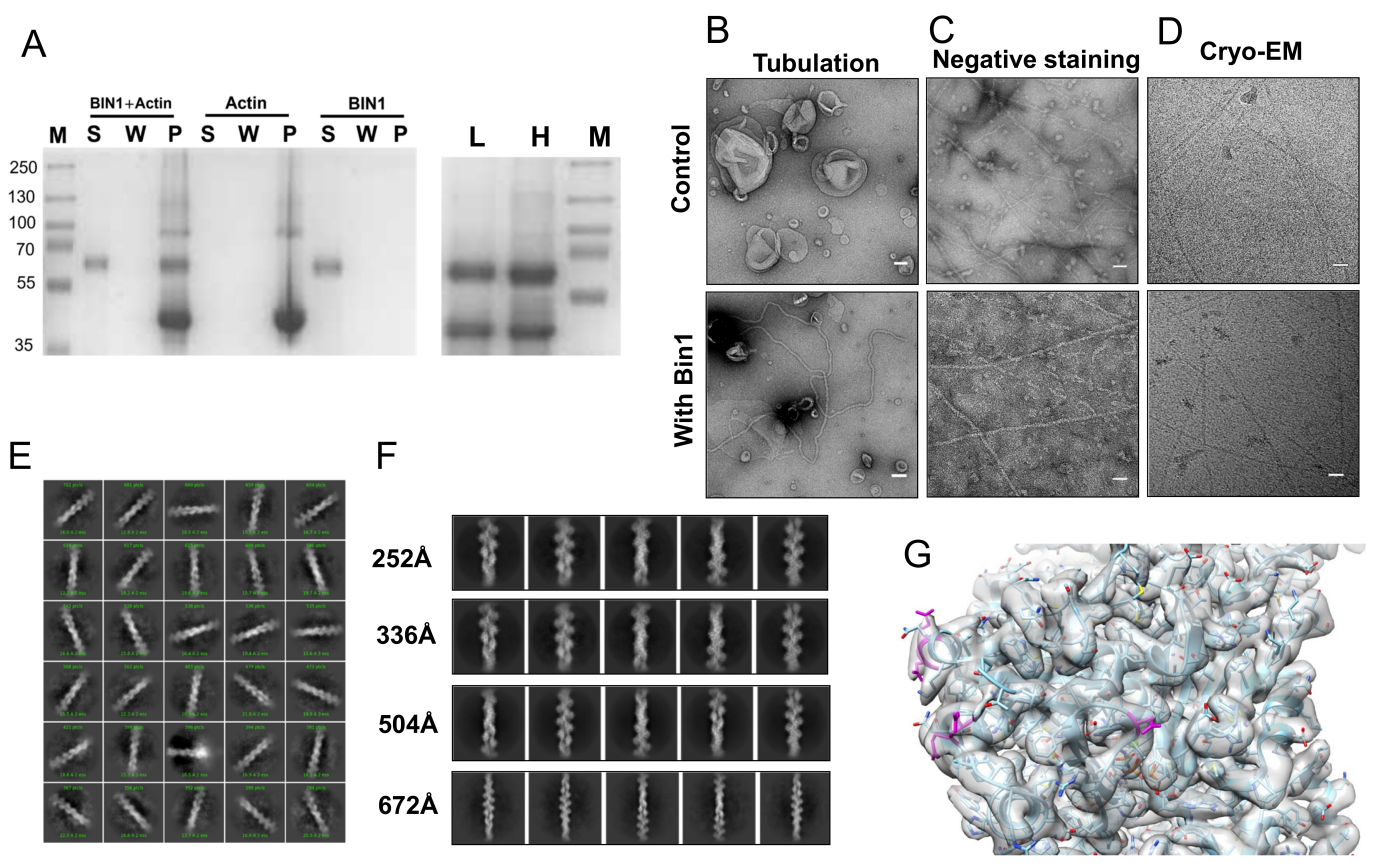
A: SDS-PAGE of a Co-sedimentation assay of BIN1 with actin filaments. M, S, W, P represents marker, supernatant, wash and pellet fractions, respectively. (left) SDS-PAGE of tested experimental conditions, showing that BIN1 can only be pelleted with actin present. (right) differential centrifugation of the BIN1-actin mix with 10,000g (L) and 100,000g (H). B: Activity verification of BIN1 by tubulation assay with liposomes (scale bar:50 nm). C: Negative staining of actin filaments alone or with BIN1(scale bar:50 nm). D: CryoEM imaging of actin filaments alone or with BIN1 (scale bar:50 nm). Images were contrast enhanced for better visibility. E: 2D classification of particles extracted from cryoEM images of actin filaments with BIN1. F: Selected 2D classed with particle box sizes of 252Å (~110000 particles), 336Å (~520000 particles), 504Å (470000 particles) and 672Å (~428000particles) show only actin filament density but no density that can be attributed to BIN1. G: 3D reconstruction (180000 particles) of the best 2D classes from the 252Å box size particle set. The pdb model of an AMPPNP bound actin filament (pdb 6djm) was fitted into the 3.25Å map. Proposed BIN1 interaction residues are in magenta.

## Description

**Description**

Bin/Amphiphysin/Rvs (BAR) domain proteins have emerged as key regulators of membrane curvature. Interestingly many BAR proteins are also steering actin dynamics during many cellular processes (Carman, 2018). The BAR protein bridging integrator 1 (BIN1) has been initially identified as part of the endocytosis and membrane trafficking machinery (Ren, 2006 ). Recently, it has been shown that BIN1 stabilizes and bundles actin filaments. In the same study, the authors reported that BIN1 influences actin dynamics. Mass spectrometry identified interaction interfaces between BIN1 and actin (Drager, 2017). We were interested to visualize the molecular details of the interaction between human BIN1 (isoform 8) and actin filaments. Therefore we collected cryo electron microscopy (cryoEM) data of actin filaments that were assembled *in vitro* and in the presence of BIN1. Curiously, we could not detect any BIN1 on single actin filaments.

We qualitatively assessed the binding between BIN1 and actin filaments through a co-sedimentation assay. BIN1 was added to G-actin at 2:1 molar ratio and left to polymerize for 30 minutes at room temperature. The reaction was then subjected to ultracentrifugation at 100,000 g for 1.5 h. A wash step was introduced to remove excess/unbound BIN1. The collected supernatant, wash and pellet fractions were analyzed by SDS PAGE (Figure 1A). Our data reaffirm that BIN1 was found in the pellet fraction together with actin, which consistent with a previous report (Drager, 2017). We also tested the presence of BIN1 on bundles with differential centrifugation of the sample; first at 10,000g for 10 min (Figure 1 A lane L) then at 100,000g as described above (Figure 1 A lane H). This experiment confirms the presence of BIN1 on bundles. To ensure that BIN1 is functional, we tested its ability to form tubes *in vitro* (Gowrisankaran, 2020). We confirmed negatively stained lipid nanotubes in electron micrographs (Figure 1B). Next we evaluated the *in vitro* formation of actin filaments by negative stain EM and cryoEM (Figure 1C and Figure 1D). The apparent diameter of ~7nm of the observed filaments is within the range of single actin filaments. The images of actin filaments in the presence of BIN1 did not differ much in negative stain or in cryoEM (Figure 1D). Yet, based on the sedimentation assay we concluded that there is an actin-BIN1 complex. For structure analysis, we collected a data set of a BIN1-actin complex without removing BIN1 from the reaction to ensure maximum coverage of actin filaments. We processed the movies and images with the CryoSparc software suite. After filament tracing and particle extraction, we performed unsupervised 2D classification (Figure 1E). Based on the X-ray structures of BIN1 and other N-BAR domains we know that BIN1’s BAR domain spans ~150 Å (Casal, 2006). Therefore we chose an initial box size of 294 Å for particle extraction. Yet, no density other than actin was observed in the 2D classes (Figure 1 E+F). If BIN1 is not bound in a 1:1 stoichiometry, we need to investigate longer stretches of the actin filament to cover larger segments. So, we increased the particle box size (and the binning). However, neither of the 2D classes showed any evidence for the presence of BIN1 (Figure 1F). Last we reconstructed actin filaments from the best 336Å box size 2D classes (180008 particles) to 3.25 Å. This resolution allowed us to unambiguously fit a model of actin. We could identify the acidic residues that were proposed to partake in the BIN1 interaction (Figure 1G, magenta). We could not identify any excess density at these residues that may indicate the presence of an interaction partner. Even, if BIN1 is flexible, we would have expected that the contact interface between BIN1 and actin would have been somewhat inflexible.

Taken together, our data confirms that BIN1 binds to actin. However, this study clarifies some of previous reports. Namely, BIN1 does not bind to single actin filaments. Instead we speculate that BIN1 can be found on actin bundles with various single filament numbers, which our differential centrifugation experiments indicate. This is in line with the cited study, where it was reported that BIN1 bundles actin filaments (Drager, 2017). Interestingly, a recent report shows that another N-BAR protein (ASAP1) can create polar bundles with a low filament number (Chen, 2020). Other BAR domain proteins (PACSIN2) are found on single filaments (Kostan, 2014). Therefore, it remains an open question if BAR domains can differentiate between single actin filaments and actin bundles.

## Methods

BIN1 construct was kindly provided by P. De Camilli and purified as previously described (Gowrisankaran, 2020). Rabbit actin was kindly provided by Dr. De La Cruz and purified as described (Elam, 2017). For the actin filament co-sedimentation assay, 30 µM BIN1 was incubated with 15 µM G-actin in polymerization buffer for 30 minutes at room temperature, as described in (Elam, 2017). The reaction was then subjected to centrifugation using TLA 100.2 fixed angle centrifuge rotor (Beckman) at 100000g for 1.5 hours. The pellets were further resuspended using the same volume of polymerization buffer for washing. The resuspended complexes were pelleted as described above. The supernatant, wash and pellets were then subjected to SDS PAGE and detected by Coomassie blue stain. For the differential centrifugation the mix was first centrifuged at 10,000g with a tabletop centrifuge. The supernatant was then transferred and spun at 100,000g as described above. Both, actin alone and actin co-polymerized with BIN1 were visualized by electron microscopy. For negative stain, actin filaments and binding reaction were diluted by 10 times and then immediately stained with 2% uranyl acetate and imaged on a JEM 2100f transmission electron microscope (JEOL). Electron micrographs were contrast enhanced for visibility. For the tubulation assay, Folch lipid fraction I (SigmaAldrich) was dissolved in chloroform and then dried under a steady stream of nitrogen and desiccated. The liposomes were formed by rehydration of the dried lipids with buffer (50mM KCl, 25 mM Tris/HCl pH 7.5) and adjusted to a stock of 1 mg/ml. For the tubulation assay 0.4 µM BIN1 was incubated with 500 µM liposomes for 30 mins at room temperature. For cryoEM, the BIN1-actin samples were directly frozen on a Cu300 2/2 grid (Quantifoil) in a Vitrobot (ThermoFisher) at 16 °C at 100% humidity with liquid ethane. Data were collected on a Titan Krios (ThermoFischer) with a K3 camera (GATAN) with a pixelsize of 0.84Å and a total dose of 50 electrons/Å^2^. Movie correction was performed with MotionCorr2 (Zheng, 2017) and the CTF estimation with CTFFIND4 (Rohou, 2015) Helical reconstruction was performed in CryoSparc3.2 (Punjani, 2017) with a helical rise of 27.657Å and a helical twist of -166.594. The map was deposited in the Electron Data Base with the accession code EMD-12762. The rigid body fitting of the pdb (6djm) was performed in the UCSF Chimera package (Pettersen, 2004).

## Reagents

**Table:** 

**Plasmid**	**Describe**	**Source**
pGEX-6P-1	The expression vector for hBin1 (isoform 8)	DeCamilli Lab
		
**Expression strain**	**Describe**	**Source**
E. coli BL21	Expression strain for BIN1	New England Biolabs
		
**Other reagents**	**Describe**	**Source**
PreScission protease	Protease to remove GST tag	GE Healthcare.
Uranyl acetate	Negative staining	SigmaAldrich
Folch fraction I	For making Folch liposomes, Brain Extract from bovine brain Type I.	SigmaAldrich
rabbit actin	Isolated from rabbit muscle, polymerization buffers provided	De La Cruz Lab
